# Transillumination of the Nasal Accessory Cavities

**Published:** 1911-02-18

**Authors:** 


### Laryngology and Rhinology.

### TRANSILLUMINATION OF THE NASAL ACCESSORY CAVITIES.

The most certain basis of diagnosis in cases of suppuration within the accessory sinuses of the nose is to see a streak of pus within the naris and to trace it to its origin in the affected cavity. But transillumination is also of decided value as an additional means of diagnosing or excluding disease. The usefulness of the method has certain limitations, and unless these are recognised the deductions drawn from the test are liable to be fallacious.

### The Method.

A small 6 to 8-volt electric lamp is usually employed, and must be provided with a rheostat to regulate the intensity of the illumination. An outer glass tube fits over the lamp and is removable for cleaning, and is replaced by a vulcanite funnel open at the end for transillumination of the frontal sinus. The room is completely darkened, or a dark cloth is thrown over the heads of patient and observer. To transilluminate the antrum, any artificial dentures are removed, the lamp is placed centrally in the mouth, the lips closed firmly over the tube, and the current turned on to appropriate strength. The light illuminates the cheeks with a red glow as high as the gingivo-labial fold. Above this the light penetrates the bones and cavities of the face in such a way as to produce certain more or less well-defined patches or *taches* of light. A patch on the side of the bridge of the nose, the nasal *tâche*, is due to light traversing the naris, and is obliterated by plugging the nose. A very bright patch, the lachrymal *tache*, is usually seen just below the inner canthus, and is produced by light passing through both naris and antrum, for it can generally only be obliterated by plugging both these cavities. Neither of these *taches* have any diagnostic significance. The light reaches the antrum through the hard palate and penetrates the roof of the cavity into the orbit, thus producing a crescentic patch of light, the antral *tache*, along the inferior orbital margin and lower lid; this merges internally with the lachrymal *tache*, which must not be mistaken for it. In addition, on pulling down the lower eyelid the sclerotic will be seen to be illuminated; the light upon the retina sometimes produces a "red reflex" of the pupil, and the patient may experience a subjective sensation of light in the eye. All these signs are absent when the antrum is opaque, but the illumination of the cheek and the nasal and lachrymal *taches* persist.

To transilluminate the frontal sinus the vulcanite funnel is firmly applied in an upward, inward, and slightly backward direction to the junction of the roof and inner wall of the orbit and as far as possible within the orbital cavity. In all cases a ring of light with a gradually fading margin surrounds the funnel, and is due to diffusion through the soft parts superficial to the bone; it is to minimise this that the funnel is to be applied as firmly and as far back as possible. In addition, when the frontal sinus is translucent an area of illumination is seen upon the forehead with a defined and frequently a scalloped edge corresponding to the natural shape of the cavity.

### The Deductions.

Opacity of the sinuses is most frequently due to suppurative disease; neoplasms also produce opacity, and undue translucency is caused by cystic dilatation or mucocele. In cases of suppuration the opacity is the result of inflammatory thickening and hyperæmia of the lining membrane, rather than to the presence of pus in the cavity, for it persists after the sinus has been washed out or drained, and only gradually clears up with the subsidence of the inflammation. But the translucency is also impaired by undue thickness of the bony walls; if this is bilateral the results of transillumination are indecisive, but if the cavities are asymmetrical the deductions drawn from the appearances are apt to be fallacious. The method is therefore far more useful when applied to the antra, in which marked asymmetry is not common, than to the frontal sinuses, which are extremely variable in size and symmetry. It may be said at once that disease of a sinus should never be diagnosed merely from opacity on transillumination, but that nevertheless there are a considerable number of cases in which the method gives valuable information, and especially in the direction of excluding disease in one or other cavity, for a completely translucent sinus is definitely not affected by suppuration. Thus, if pus be found exuding into the middle meatus, it is often a matter of some difficulty to determine whether it proceeds from the antrum or frontal sinus, or from both these cavities; if one be found to be opaque and the other clear, the diagnosis is materially advanced. Again, if disease of the antrum is definitely established, it becomes of importance to exclude associated suppuration of the frontal sinus, and this can be done if it prove clearly translucent; if, however, it be dark and the sinus of the opposite side clear, this is not an absolute proof of suppuration, for the suspected sinus may merely be very small. Here skiagraphy is useful, for it is strongly suggestive of disease in the opaque sinus if it be shown to be as large as the translucent one of the opposite side. To sum up, opacity may be due merely to thickness of the sinus walls; suppuration does not always produce complete opacity, though it always impairs translucency; suppurative disease may be excluded in a sinus which is strongly translucent and as bright as its opposite fellow, but no definite conclusions should be drawn unless the *taches* are clear and distinct. If pus be found in the middle meatus, and the corresponding antrum is dark while its fellow is clear, it is strong presumptive evidence of antral empyema; if the corresponding frontal sinus is dark and its fellow clear, the evidence is far less certain, but may be accepted as confirmatory if skiagraphy shows that the suspected sinus is of ample size.

